# 32 A Qualitative Analysis of Elite Athletes Perceptions of Pregnancy in the United Kingdom

**DOI:** 10.1093/eurpub/ckae114.035

**Published:** 2024-09-26

**Authors:** Catherine Caro, Storm Trow, Zoë Bell, Angela Flynn, Fiona Lavelle

**Affiliations:** Royal College Of Surgeons In Ireland, Dublin, Ireland; Kings College London, London, United Kingdom; Royal College Of Surgeons In Ireland, Dublin, Ireland; Royal College Of Surgeons In Ireland, Dublin, Ireland; Kings College London, London, United Kingdom; Royal College Of Surgeons In Ireland, Dublin, Ireland

## Abstract

**Purpose:**

Female athletes train and compete at an elite level during their reproductive years, with an increasing number of athletes navigating an athletic career alongside pregnancy. There is a paucity of research exploring the antenatal experiences of elite athletes. The purpose of this study was to explore the experiences of elite athletes in the United Kingdom as they navigated pregnancy and provide considerations for future research and policy surrounding elite sport participation during pregnancy.

**Methods:**

Semi-structured online interviews were conducted in elite athletes ≥ 18 years old, who resided in the UK, and who trained and/or competed at the highest level of their sport prior to and/or during pregnancy. A topic guide was used to cover the athletes’ experiences in elite sport, specifically during preconception, pregnancy and the postpartum period. The interviews were recorded, transcribed and analysed using inductive thematic analysis. Demographic data was summarised using IBM SPSS Statistics.

**Results:**

Eleven UK athletes (mean age 31 ± 3 years) from nine team and individual sports, including sprint, endurance, ball game, power, precision and technical and combat, participated in the study. Four key themes; (1) The identity shift: athlete to mother, (2) From podium to parenthood: preconception and pregnancy planning, (3) Is that my career over?: upon becoming pregnant (4) Navigating the bump: elite training and pregnancy, were developed. The participants spoke about the intrinsic and extrinic challenges, decisions and considerations related to maternal health and elite sport. The experiences described the shifting paradigm of being an athlete and becoming a mother, physical and mental health changes, adapting training, support networks and gaps in knowledge, research and guidance around pregnancy and elite sport.

**Conclusions:**

This study highlights the challenges elite athletes encounter in balancing an athletic career with motherhood. The findings support policy and research recommendations, advocating for improved access to physical and mental health support, modified training behaviours, tailored nutrition guidance, increased preconception health awareness and maternity funding to enhance inclusivity and better support elite athletes during pregnancy.

